# Development of a short-term prognostic model for hepatitis B-related acute-on-chronic liver failure in a dual-center cohort

**DOI:** 10.3389/fmed.2025.1688479

**Published:** 2026-01-12

**Authors:** Huaidong Deng, Ruili Bao, Yanxue Lu, Hui Xu, Jia Yao, Rongkuan Li

**Affiliations:** 1 Department of Infectious Disease, The Second Hospital of Dalian Medical University, Dalian, China; 2 Department of Gastroenterology, Shanxi Bethune Hospital, Taiyuan, China; 3 Department of Nephrology, The Second Hospital of Dalian Medical University, Dalian, China

**Keywords:** acute-on-chronic liver failure, hepatitis B virus, interleukin-6, prognosis, SIRS

## Abstract

**Background and aims:**

Acute-on-chronic liver failure (ACLF) is a severe clinical syndrome marked by rapid progression and high short-term mortality. In the Asia-Pacific region, where the hepatitis B virus (HBV) is endemic, HBV-related ACLF (HBV-ACLF) represents the most common subtype. The aim of this study was to develop and validate a concise and accurate nomogram for predicting 28-day mortality in patients with HBV-related ACLF.

**Methods:**

A total of 159 patients with HBV-ACLF were enrolled, including 113 in the training cohort and 46 in the validation cohort. Clinical characteristics, routine laboratory parameters, and inflammatory cytokine levels were collected. In the training cohort, least absolute shrinkage and selection operator (LASSO) regression was applied to select variables, followed by multivariate logistic regression to construct a nomogram model (ICI). Model performance was evaluated in both cohorts using receiver operating characteristic (ROC) curves, calibration plots, and decision curve analysis (DCA), and compared with COSSH-ACLF II, CLIF-C ACLF, AARC, CLIF-OFs, MELD, and CTP scores.

**Results:**

LASSO regression identified ln(INR), ln(Cr), ln(WBC), and ln(IL-6) as candidate predictors. Multivariate logistic regression further confirmed that ln(INR), ln(Cr), and ln(IL-6) were independent risk factors. The new model (ICI) demonstrated good discriminative ability with an area under the receiver operating characteristic curve (AUC) of 0.826 in the training cohort and 0.814 in the validation cohort. Calibration analysis showed excellent consistency, and decision curve analysis (DCA) indicated that the ICI model provided higher net clinical benefit across different threshold probabilities. Furthermore, compared with traditional models, the ICI model exhibited significant advantages in terms of discriminative ability and clinical benefit.

**Conclusion:**

The newly developed model (ICI) showed superior predictive performance for short-term prognosis in patients with HBV-ACLF, outperforming conventional scoring systems. It is anticipated that the ICI model will serve as a valuable instrument in complementing the conventional scoring system for the HBV-ACLF population in the future.

## Introduction

Acute-on-chronic liver failure (ACLF) is a clinical syndrome characterized by rapid progression to hepatic decompensation occurring in patients with underlying chronic liver disease or cirrhosis, following acute precipitating events such as infections, variceal bleeding, or drug-induced liver injury. ACLF is marked by rapid deterioration of liver function, often accompanied by multiple organ dysfunction syndrome (MODS), manifesting significant clinical heterogeneity and extremely high short-term mortality rates (1–3). Due to its rapid progression, unpredictable organ failure trajectory, and narrow therapeutic window, ACLF is widely recognized as one of the most challenging critical conditions in hepatology (4). The rising prevalence of chronic liver diseases has correspondingly increased the incidence of ACLF annually (5, 6). Globally, ACLF occurs in approximately 35% of patients with decompensated cirrhosis (7), and a retrospective cohort study reported a 28-day mortality rate of about 40% in ACLF patients (8). Its dual characteristics of high incidence and mortality have progressively made ACLF a major public health challenge worldwide. In Asian regions, particularly China, hepatitis B virus (HBV) infection remains the primary etiology, accounting for the majority of ACLF cases (9, 10).

In terms of pathogenesis, current research indicates systemic inflammation and immune dysregulation as key drivers in ACLF onset and progression. Acute insults rapidly activate the hepatic innate immune response, leading to elevated proinflammatory cytokines, such as interleukin-6 (IL-6) and tumor necrosis factor-alpha (TNF-*α*), forming a sustained and intense cytokine release state (“cytokine storm”). This process not only exacerbates hepatocyte necrosis but also significantly contributes to MODS through mechanisms including increased vascular permeability and disrupted microcirculatory perfusion (11–13).

Currently, several prognostic scoring systems for ACLF have been widely utilized in clinical practice, including MELD (14), Child-Turcotte-Pugh (CTP) (15), CLIF-Organ Failure score (CLIF-OFs) (16), CLIF Consortium ACLF (CLIF-C ACLF) (17), Asian Pacific Association for the Study of the Liver ACLF Research Consortium (AARC) (2), and the Chinese Group on the Study of Severe Hepatitis B COSSH-ACLF II (18). MELD and CLIF-C scoring systems were developed primarily from European and American populations, predominantly comprising white patients with alcohol-related liver disease and hepatitis C virus infection. Significant differences exist in etiology and population characteristics compared with HBV-dominated ACLF populations in Asia, thus limiting their generalizability and predictive performance in HBV-ACLF patients. Although the COSSH scoring system was developed specifically for Chinese HBV-ACLF patients and is widely used, its predictive model relies heavily on COSSH-defined ACLF diagnostic criteria, which emphasize existing organ failure and a higher total bilirubin threshold (TBil ≥12 mg/dL). Compared to the broader APASL criteria (TBil ≥5 mg/dL), the COSSH system tends to identify patients in mid-to-late disease stages (2, 18, 19). Consequently, it has limitations in identifying and stratifying early-stage HBV-ACLF patients, thus restricting its applicability for early therapeutic interventions.

This study aims to construct a concise and practical 28-day mortality risk prediction model integrating routine laboratory indicators and inflammatory cytokines in HBV-ACLF patients diagnosed under the APASL criteria. By comparing its performance with conventional scoring systems, this model aims to enhance early risk stratification and support clinical decision-making.

## Methods

### Study population

**Figure 1:**
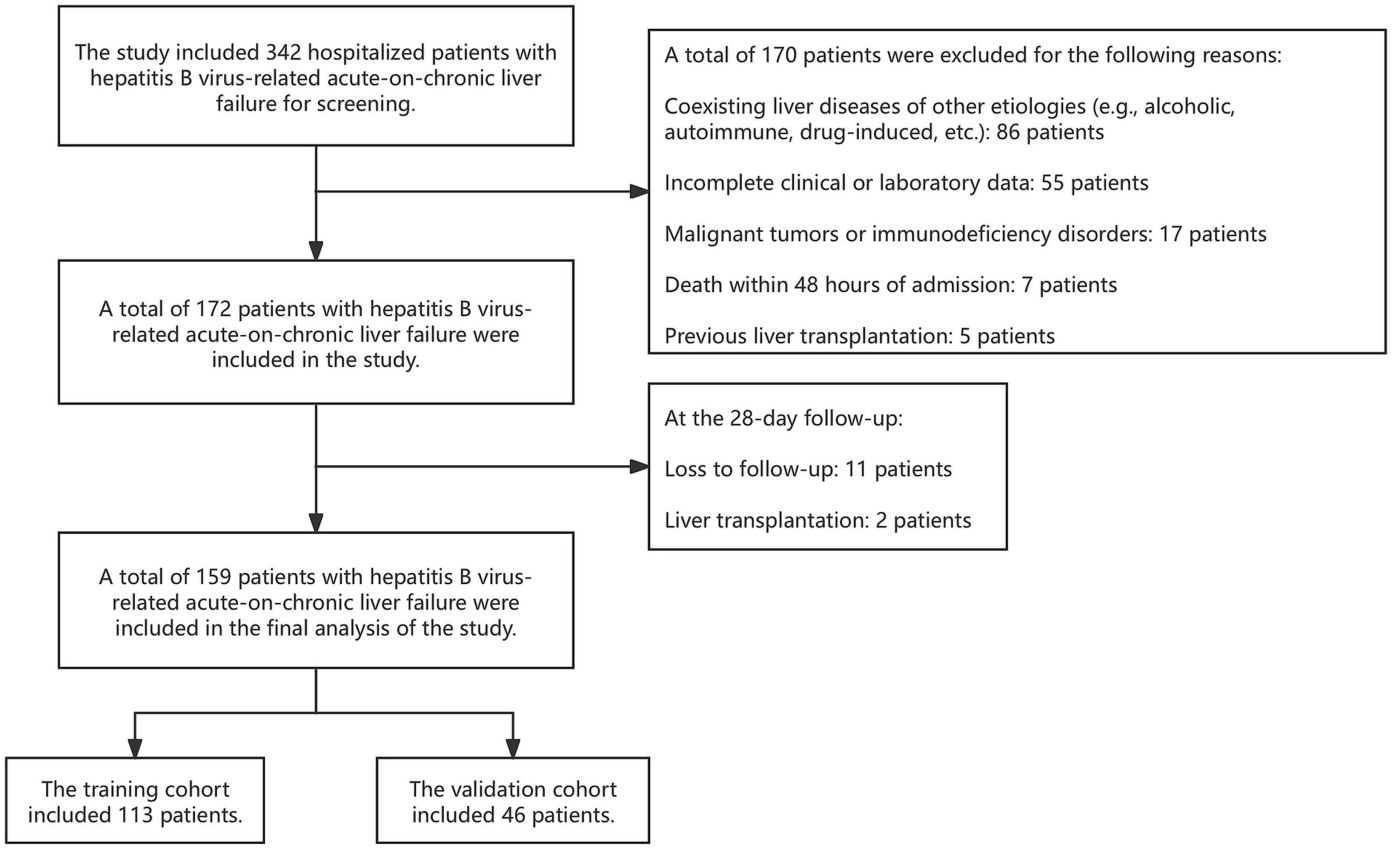
Flowchart of screening, recruitment of patients with HBV-ACLF.

This study was a dual-center retrospective cohort study. Patients with HBV-ACLF who met the inclusion criteria from January 2020 to January 2025 at the Second Affiliated Hospital of Dalian Medical University were enrolled as the training cohort (n = 113). Additionally, eligible patients from Shanxi Bethune Hospital recruited between March 2022 and September 2024 were designated as the validation cohort (n = 46), a total of 159 patients were included in the final analysis (Figure 1).

The study was conducted in accordance with the ethical principles outlined in the Declaration of Helsinki and received approval from the Ethics Committee of the Second Affiliated Hospital of Dalian Medical University (approval number: KY2025-485-01). In light of the retrospective nature of the study, the requirement for written informed consent was waived by the Ethics Committee.

### Inclusion and exclusion criteria

Inclusion criteria were as follows:

1. Age ≥18 years;
2. Confirmed chronic hepatitis B virus (HBV) infection (20);
3. Diagnosis of ACLF based on the criteria of the Asian Pacific Association for the Study of the Liver (APASL) (2);
4. Completion of key laboratory tests within 48 h of admission.

Exclusion criteria included:

1. Coexisting liver diseases of other etiologies (e.g., alcoholic, autoimmune, drug-induced, etc.);
2. Presence of malignant tumors or immunodeficiency disorders;
3. History of liver transplantation;
4. Death within 48 h of admission;
5. Incomplete clinical or laboratory data.

### Data collection

Clinical data were extracted from the hospital’s electronic medical records system within 48 h of patient admission, including age, sex, body mass index (BMI), and blood pressure. Laboratory parameters assessed included white blood cell count, hemoglobin, platelet count, serum albumin, alanine aminotransferase (ALT), aspartate aminotransferase (AST), total bilirubin, serum creatinine, serum sodium, international normalized ratio (INR), and inflammatory cytokines, among others. Additionally, complications such as hepatic encephalopathy, infection, ascites, electrolyte disturbances, and whether the patient received artificial liver support therapy were documented.

All enrolled patients were followed from the day of admission until death or the end of the 28-day follow-up period to assess short-term outcomes. Survival status was confirmed through both electronic medical records and telephone follow-up.

### Calculation of conventional ACLF scores

The following established prognostic scores were calculated for all enrolled patients: MELD = 3.78 × ln [TBil (mg/dL)] + 11.2 × ln [INR] + 9.57 × ln [Creatinine (mg/dL)] + 6.43 (14); CLIF-C ACLF = 10 × [0.33 × CLIF-OFs + 0.04 × age + 0.63 × ln (WBC count) – 2] (17); COSSH-ACLF II = 1.649 × ln (INR) + 0.457 × grade of hepatic encephalopathy (1: grade 0; 2: grade I–II; 3: grade III–IV) + 0.425 × ln [neutrophil count (×10^9^/L)] + 0.396 × ln [TBil (μmol/L)] + 0.576 × ln [blood urea (mmol/L)] + 0.033 × age (18); AARC (2); CTP (15); CLIF-OFs (16).

### Data preprocessing

Before constructing the predictive model, normality tests were performed on all continuous variables using the Shapiro–Wilk test to assess the distribution of the data. For variables that significantly deviated from normality, a natural logarithm transformation was applied to make the data more normal-looking, thereby better satisfying the assumptions of statistical models such as linear regression. Based on the transformed data, Pearson correlation coefficients were calculated to evaluate the correlations between pairs of variables, and a correlation heatmap was generated. For pairs of variables with absolute correlation coefficients greater than 0.7, one variable from each pair was retained for subsequent analysis. Factors such as clinical relevance and generalisability were considered in order to reduce redundancy and minimize the risk of multicollinearity, thereby enhancing the stability of the model.

### Model development

In the training cohort, candidate variables were selected using the least absolute shrinkage and selection operator (LASSO) regression, with the optimal penalty parameter (*λ*) determined through 10-fold cross-validation. These variables were then incorporated into a multivariate logistic regression model and a nomogram was constructed to predict 28-day mortality based on the regression results for variables with a *p*-value less than 0.05.

### Model performance evaluation and comparison

The performance of the model was comprehensively assessed in both the training and validation cohorts. Discriminatory ability was evaluated by calculating the area under the receiver operating characteristic (ROC) curve (AUC). The model’s calibration was assessed using several metrics, including the Brier score, calibration curve, calibration slope, and calibration intercept. Additionally, the clinical utility of the model was evaluated through decision curve analysis (DCA) to estimate the net benefit across various threshold probabilities. To further validate the model’s effectiveness, this study systematically compared the predictive performance of the ICI model with six conventional scoring systems in both cohorts. The comparison was conducted using ROC curves, net reclassification improvement (NRI), integrated discrimination improvement (IDI), probability density function (PDF) analysis, and clinical decision curve analysis.

### Statistical analysis

All statistical analyses were performed using R software (version 4.3.3). The primary R packages used were glmnet, rms, pROC, ggplot2 and rmda. The normality of continuous variables was assessed using the Shapiro–Wilk test. For normally distributed data, the mean ± standard deviation was reported and compared using a t-test. For non-normally distributed data, the median (interquartile range) was reported and compared using the Wilcoxon rank-sum test. Categorical variables were presented as frequencies (percentages) and compared using the chi-squared test (when the expected frequency was greater than or equal to 5) or Fisher’s exact test (when the expected frequency was less than 5). LASSO regression with 10-fold cross-validation was used for variable selection. The selected variables were then incorporated into a multivariate logistic regression model to construct a nomogram. The model’s performance was evaluated in both the training and validation cohorts using methods including receiver operating characteristic (ROC) curves, calibration plots and decision curve analysis (DCA). The model’s performance was then compared with that of six conventional scoring systems: COSSH-ACLF II, CLIF-C ACLF, MELD, AARC, CLIF-OFs and CTP. All hypothesis tests were two-sided and a *p*-value of less than 0.05 was considered statistically significant.

## Results

### Baseline characteristics

The mean age of the training cohort was 54.92 years (SD 11.35) and 32.7% of patients were female. The incidence rates for hepatic encephalopathy (HE), ascites, electrolyte imbalance (EI), infection, hepatorenal syndrome (HRS) and hepatopulmonary syndrome (HPS) were 60.2, 75.2, 60.2, 59.3, 23.9 and 15.0%, respectively. The baseline characteristics of patients in the validation cohort were largely similar to those in the training cohort, with no significant differences observed for most variables (*p* > 0.05). Detailed information is provided in Table 1.

**Table 1:** Baseline clinical characteristics.

Baseline characteristics	Training cohort (*n* = 113)	Validation cohort (*n* = 46)	*p*-value
Age in years	54.92 ± 11.35	51.74 ± 11.93	0.054
Sex, female (%)	37 (32.7)	11 (23.9)	0.363
HE, yes (%)	68 (60.2)	17 (37.0)	0.013
Ascites, yes (%)	85 (75.2)	35 (76.1)	1.000
EI, yes (%)	68 (60.2)	17 (37.0)	0.013
Infection, yes (%)	67 (59.3)	30 (65.2)	0.606
HRS, yes (%)	27 (23.9)	5 (10.9)	0.101
HPS, yes (%)	17 (15.0)	4 (8.7)	0.416
ALSS, yes (%)	60 (53.1)	30 (65.2)	0.222
SBP in mmHg	120.00 [113.00, 128.00]	127.00 [113.50, 138.75]	0.047
DBP in mmHg	74.55 ± 11.56	75.13 ± 13.89	0.787
MAP in mmHg	90.00 [83.00, 96.33]	92.33 [85.83, 102.75]	0.154
BMI in kg/m^2^	25.64 [23.66, 27.78]	25.95 [22.42, 27.72]	0.658
TBil in μmol/L	231.62 [107.70, 337.66]	242.35 [165.32, 412.18]	0.126
ALB in g/L	28.39 ± 5.94	29.07 ± 4.74	0.489
ALT in U/L	235.50 [34.43, 1005.84]	276.05 [53.80, 1089.90]	0.092
AST in U/L	125.80 [66.52, 328.60]	156.05 [76.35, 378.02]	0.137
PT in s	22.50 [20.10, 26.70]	23.25 [19.15, 29.00]	0.766
PTA in %	38.69 ± 11.56	35.09 ± 12.57	0.084
INR	1.99 [1.72, 2.36]	2.13 [1.77, 2.68]	0.122
CR in μmol/L	72.45 [60.00, 85.00]	77.10 [69.50, 90.00]	0.075
WBC as ×10^9^/L	6.30 [4.06, 9.20]	6.00 [4.53, 10.70]	0.758
CRP in mg/L	18.99 [10.77, 35.06]	13.29 [6.62, 23.90]	0.035
HB in g/L	112.00 [92.00, 134.00]	125.50 [111.00, 139.25]	0.003
PLT as ×10^9^/L	74.00 [45.00, 118.00]	90.00 [56.75, 130.75]	0.167
NEU as ×10^9^/L	4.53 [2.59, 7.14]	4.22 [3.03, 7.82]	0.976
LYM as ×10^9^/L	0.83 [0.54, 1.21]	0.92 [0.64, 1.36]	0.287
NLR	5.44 [2.61, 10.32]	5.00 [3.19, 8.31]	0.849
Lac in mmol/L	2.90 [2.00, 4.10]	2.68 [1.91, 3.70]	0.511
AFP in ng/mL	27.12 [3.30, 105.83]	13.75 [6.43, 83.00]	0.921
K in mmol/L	3.91 [3.47, 4.24]	3.84 [3.43, 4.23]	0.669
Na in mmol/L	135.84 [133.20, 138.20]	134.45 [132.40, 136.78]	0.081
NH3 in μmol/L	47.00 [31.00, 65.00]	30.55 [25.30, 42.77]	0.010
IL-6 in pg./mL	26.58 [15.33, 62.18]	19.71 [9.02, 43.31]	0.100
IL-8 in pg./mL	98.40 [55.10, 173.00]	64.89 [36.12, 148.35]	0.226
IL-1β in pg./mL	9.19 [5.21, 16.40]	8.27 [4.59, 15.36]	0.158
TNF-α in pg./mL	23.40 [16.60, 50.70]	22.82 [14.03, 43.75]	0.0843
Log(DNA) in Log(IU/mL)	4.85 [2.59, 7.45]	5.42 [4.25, 6.53]	0.171
CLIF-C	45.76 ± 10.26	47.80 ± 8.10	0.229
AARC	9.00 [8.00, 9.50]	9.50 [8.50, 10.50]	0.053
MELD	21.50 [19.00, 24.00]	23.50 [21.00, 27.00]	0.068
COSSH-ACLF II	8.20 [7.50, 9.00]	8.00 [7.60, 8.40]	0.125
CTP	11.00 [10.00, 13.00]	10.50 [9.00, 12.00]	0.016
CLIF-OFs	9.00 [8.00, 11.00]	10.02 [9.48, 11.18]	0.070

SBP, systolic blood pressure; DBP, diastolic blood pressure; MAP, mean arterial pressure; Ht, height; Wt, weight; BMI, body mass index; HE, hepatic encephalopathy; EI, electrolyte imbalance; HRS, hepatorenal syndrome; HPS, hepatopulmonary syndrome; ALSS, artificial liver support system; TBil, total bilirubin; Alb, albumin; NLR, neutrophil-to-lymphocyte ratio; ALT, alanine aminotransferase; AST, aspartate aminotransferase; PT, prothrombin time; PTA, prothrombin activity; INR, international normalized ratio; Cr, creatinine; WBC, white blood cell count; CRP, C-reactive protein; Hb, hemoglobin; NEU, neutrophil count; PLT, platelet count; LYM, lymphocyte count; Lac, lactate; AFP, alpha-fetoprotein; K, serum potassium; Na, serum sodium; NH3, ammonia; IL-6, interleukin-6; IL-8, interleukin-8; IL-1β, interleukin-1β; TNF-α, tumor necrosis factor-α; IL-10, interleukin-10; Log(DNA), log(hepatitis B virus DNA viral load); CLIF-C, the Study of acute-on-chronic liver failure score; MELD, Model for end-stage liver disease score; COSSH-ACLF II, Chinese group on the study of severe hepatitis B-ACLF II score; CTP, Child-Turcotte-Pugh score; CLIF-OFs, chronic liver failure organ failure score.

### Data preprocessing

**Figure 2:**
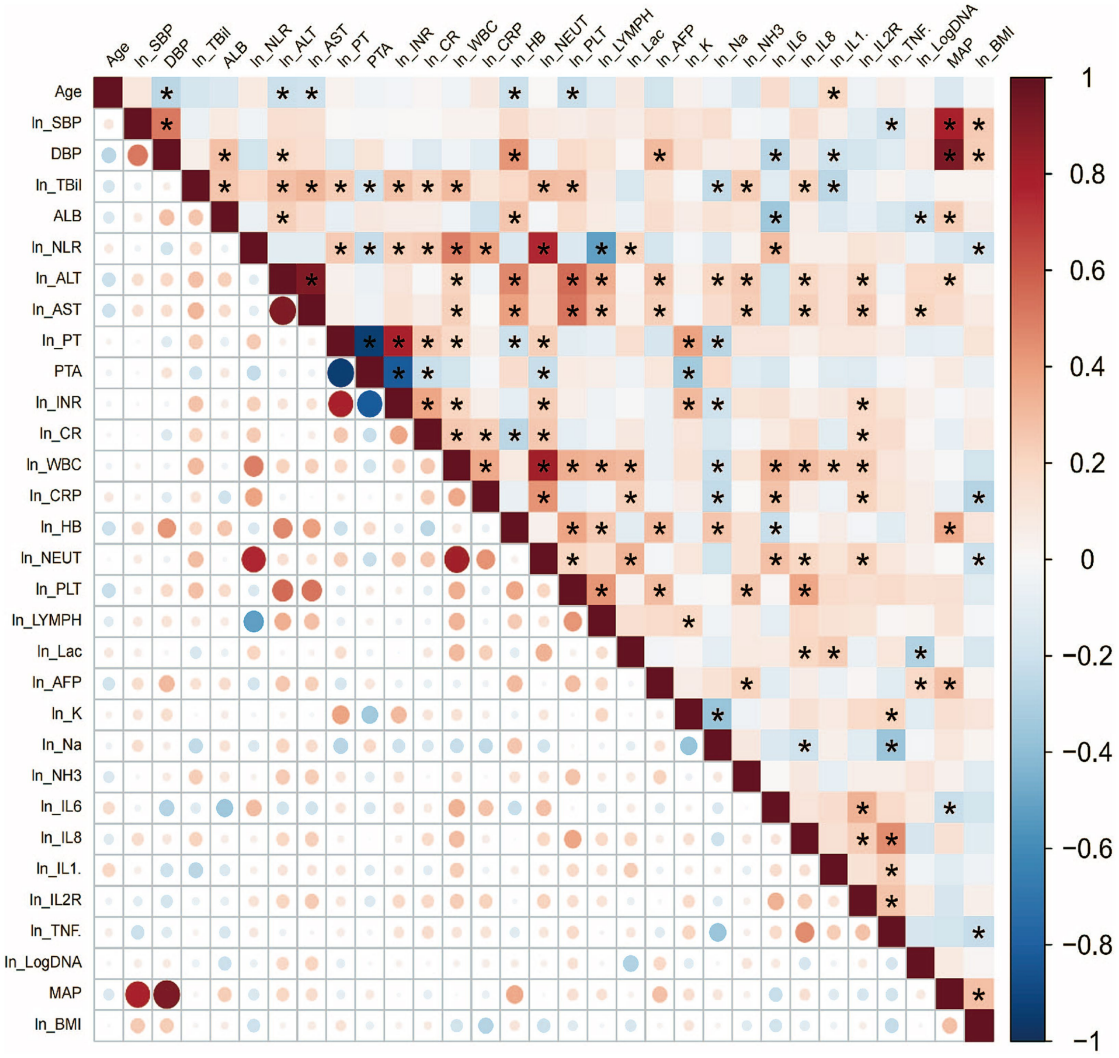
Correlation heatmap of clinical variables. The heatmap shows the correlation between variables, where red indicates strong positive correlation, blue indicates strong negative correlation, and white indicates no correlation. Asterisks (*) represent statistically significant correlations (*p* < 0.05).

In the training cohort, Pearson correlation coefficients were calculated for the candidate variables and a correlation heatmap was generated (Figure 2). This analysis revealed significant linear correlations between several variables, including ln(ALT) and ln(AST) (*r* = 0.911, *p* < 0.01), ln (PT) and PTA (*r* = −0.938, *p* < 0.01), DBP and MAP (*r* = 0.930, *p* < 0.01) and ln(NLR) and ln(NEUT) (*r* = 0.756, *p* < 0.01). These results suggest the presence of multicollinearity. Based on clinical relevance and generalisability, one variable from each highly correlated pair was retained and the redundant variables were excluded. For instance, both ALT and AST are biochemical markers of hepatocellular injury; however, since ALT is more specific to the liver and reflects the degree of liver damage in hepatitis B-related liver failure more accurately, it was selected as the model variable while AST was excluded. Following this selection process, seven redundant variables were removed, leaving 34 variables for subsequent LASSO regression analysis.

### Variable selection and model construction

**Figure 3:**
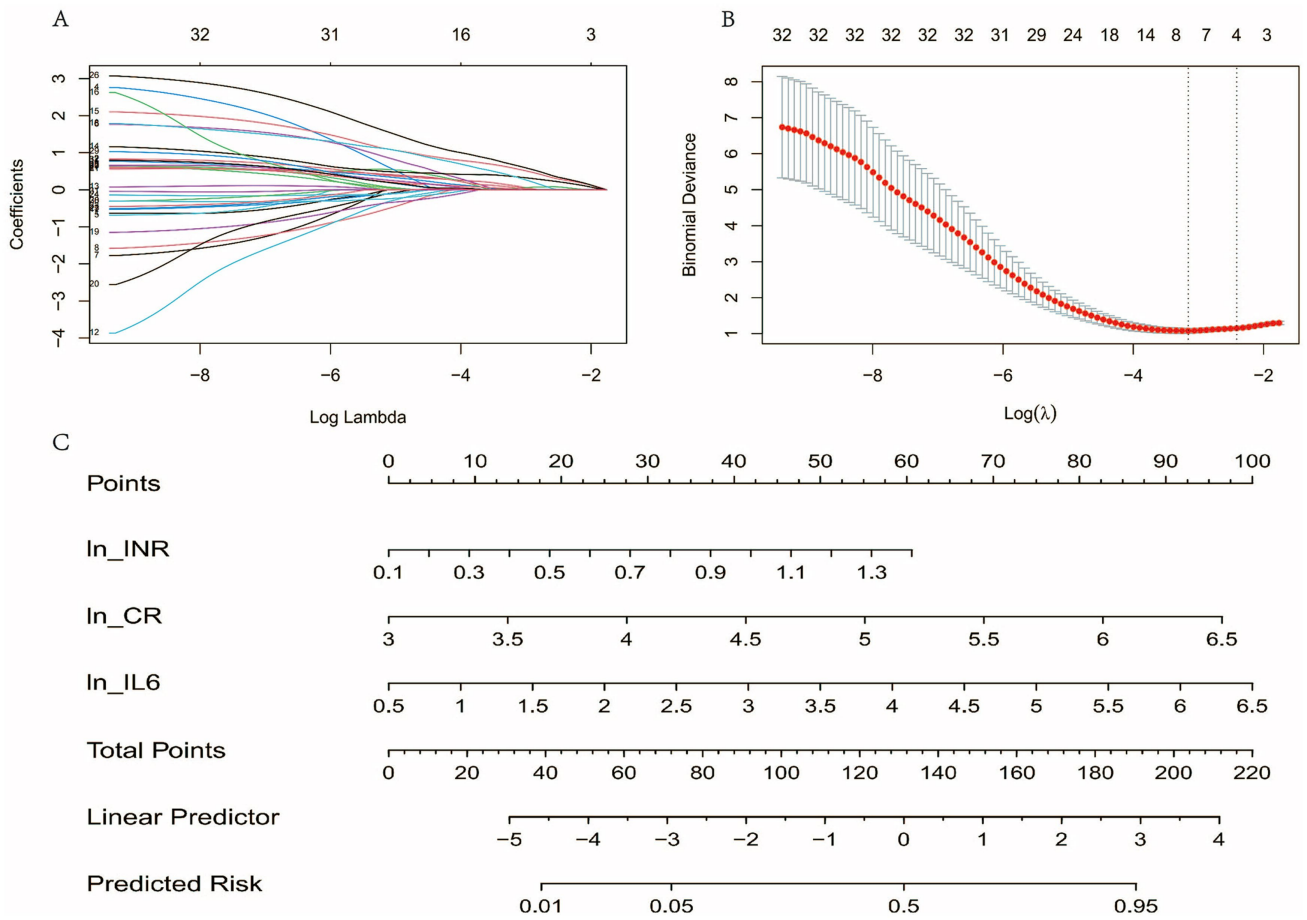
Lasso regression and nomogram for predicting risk. **(A)** Coefficients of predictors across different values of log(*λ*). **(B)** Binomial deviance plot for selecting optimal λ. **(C)** Nomogram for predicting risk, showing points for each predictor (ln_INR, ln_CR, ln_IL6), total points, linear predictor, and predicted risk. “ln_” indicates the natural logarithm transformation of the variable.

In the training cohort, 10-fold cross-validation combined with LASSO regression analysis was applied to select from 34 candidate variables. Using the optimal penalty parameter (*λ* = 0.08958842), four variables with non-zero coefficients were identified: ln(INR), ln(CR), ln(WBC), and ln(IL-6) (Figures 3A,B). These variables were subsequently included in a multivariate logistic regression analysis. The results indicated that ln(INR) [O*R* = 1.7259, 95% CI (1.0241, 2.9087), *p* = 0.0404], ln(CR) [O*R* = 1.9799, 95% CI (1.1735, 3.3406), *p* = 0.0105], and ln(IL-6) [O*R* = 2.3684, 95% CI (1.3262, 4.2298), *p* = 0.0036] were independent predictors of 28-day mortality, whereas ln(WBC) [O*R* = 1.2998, 95% CI (0.7572, 2.2312), *p* = 0.3416] did not show statistical significance (Table 2). Based on these findings, a predictive model was developed: ICI = 1.73 × ln[INR] + 1.98 × ln[Cr (μmol/L)] + 2.37 × ln[IL-6 (pg/mL)]. A nomogram (Figure 3C) was subsequently constructed to provide a visual risk assessment tool for clinical decision-making.

**Table 2:** Multivariate logistic regression results.

Variable	OR	Lower 95% CI	Upper 95% CI	*P*-value
ln_INR	1.7259	1.0241	2.9087	0.0404
ln_CR	1.9799	1.1735	3.3406	0.0105
ln_WBC	1.2998	0.7572	2.2312	0.3416
ln_IL6	2.3684	1.3262	4.2298	0.0036

OR, odds ratio; 95% CI = 95% confidence interval for the OR; statistical significance was set at *p* < 0.05. “ln_” indicates the natural logarithm transformation of the variable.

### Discrimination of the model

**Figure 4:**
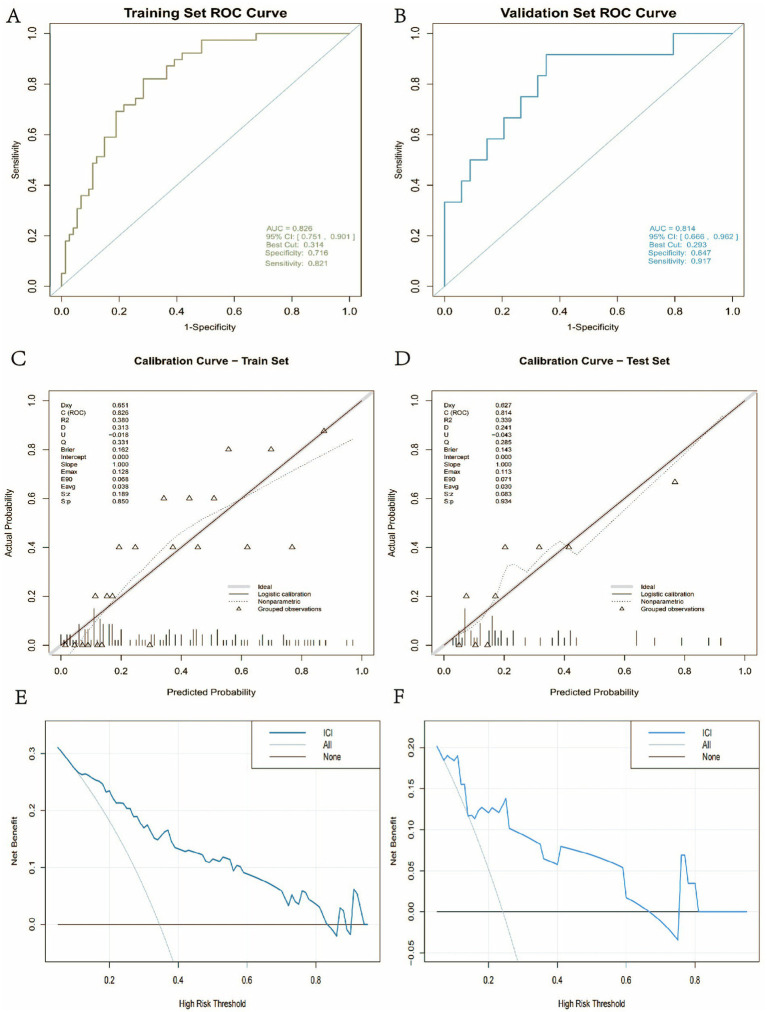
Model performance evaluation. **(A)** ROC curve for the training set; **(B)** ROC curve for the validation set; **(C)** calibration curve for the training set; **(D)** calibration curve for the test set; **(E)** decision curve analysis (DCA) for the training set; **(F)** decision curve analysis (DCA) for the test set.

To evaluate the ICI model’s discriminative ability, receiver operating characteristic (ROC) curves were plotted for both the training and validation cohorts, and the area under the curve (AUC) was calculated. In the training cohort (Figure 4A), the model demonstrated good discriminatory ability, achieving an AUC of 0.826 and a 95% confidence interval (CI) of [0.751, 0.901]. At the optimal cut-off value of 0.314, the sensitivity and specificity were 0.821 and 0.716, respectively. In the independent validation cohort (Figure 4B), the AUC was 0.814, with a 95% CI of [0.666, 0.962], indicating consistent and robust predictive performance across different datasets. At the optimal threshold of 0.293, the sensitivity and specificity were 0.917 and 0.647, respectively. Taken together, these results suggest that the model demonstrates strong generalisability and robustness.

### Calibration of the ICI model

To evaluate the calibration performance of the ICI model, calibration curves were plotted for both the training and validation cohorts. In the training cohort, the calibration slope was 1.000, the intercept was 0.000, the Brier score was 0.162, and the average and maximum absolute errors (Eavg and Emax) were 0.038 and 0.128, respectively (Figure 4C), indicating a strong agreement between the predicted probabilities and the actual outcomes. In the independent validation cohort (Figure 4D), the calibration slope and intercept remained 1.000 and 0.000, respectively, while the Brier score slightly increased to 0.143. The Eavg and Emax values also improved to 0.030 and 0.113, respectively, reflecting the model’s stability across different datasets. The nonparametric calibration curve closely overlapped with the ideal 45-degree reference line, further confirming the ICI model’s excellent external calibration performance.

The Hosmer-Lemeshow test also supported the model’s good calibration in both datasets. For the training cohort, the χ^2^ statistic was 5.7411 with a *p*-value of 0.6762, and for the validation cohort, the χ^2^ statistic was 7.1307 with a p-value of 0.5226. Both *p*-values exceeded 0.05, indicating no significant difference between the predicted probabilities and actual outcomes, which corroborates the model’s calibration accuracy across both cohorts.

### Decision curve analysis of the ICI model

To further evaluate the clinical utility of the ICI model across a range of threshold probabilities, decision curve analysis (DCA) was performed to plot net benefit curves for both the training and validation cohorts (Figures 4E,F). The results demonstrated that in both cohorts, the ICI model provided favorable net clinical benefit across most threshold ranges, indicating a stable decision-making advantage under various clinical risk thresholds.

### Comparison with traditional scoring systems

**Figure 5:**
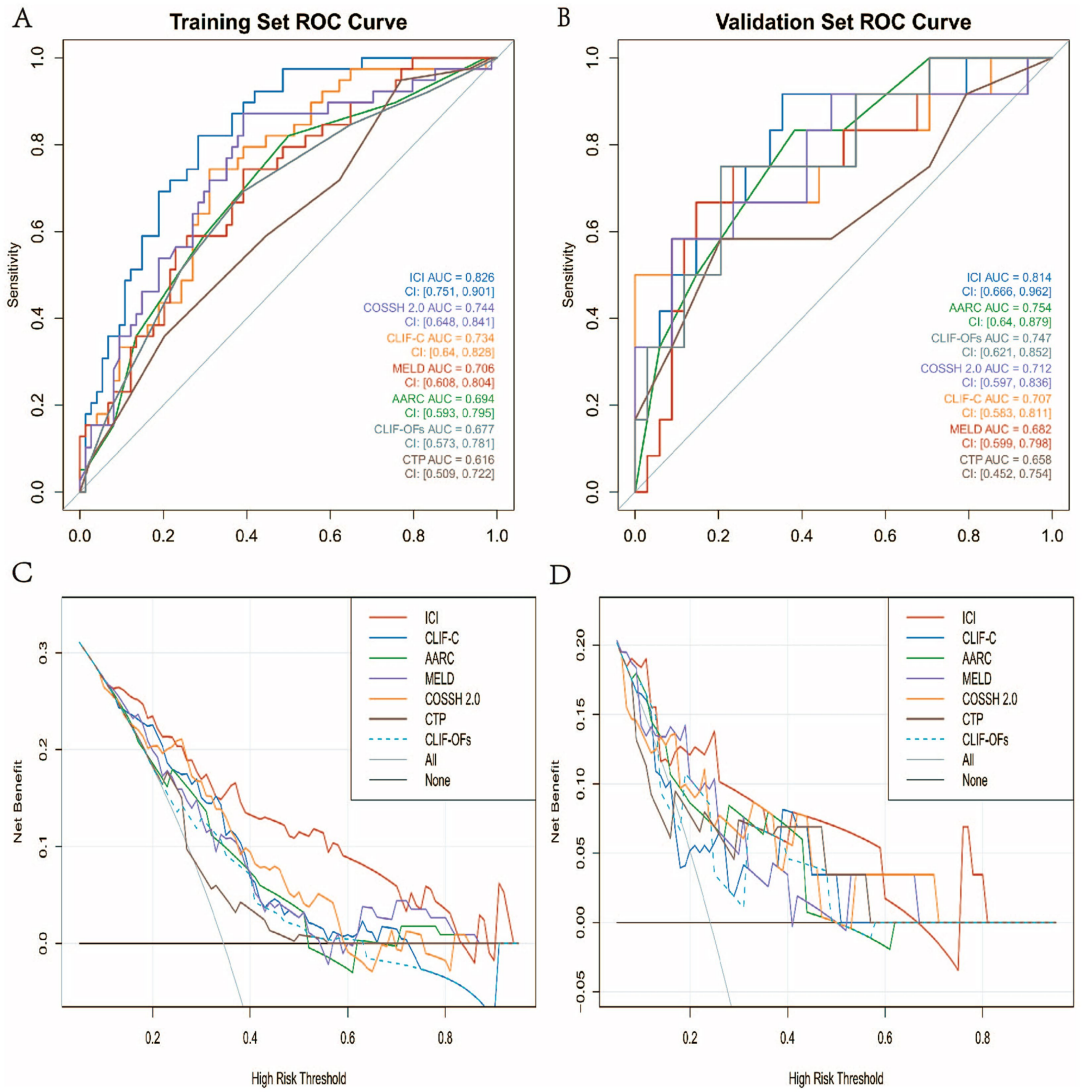
Performance evaluation of risk prediction models. Panels **(A,B)** display the ROC curves for the training and validation sets, respectively, comparing multiple models with their corresponding AUC values. Panels **(C,D)** present the decision curve analysis (DCA) for the training and test sets, respectively, illustrating the net benefit of each model at various high-risk thresholds.

**Figure 6:**
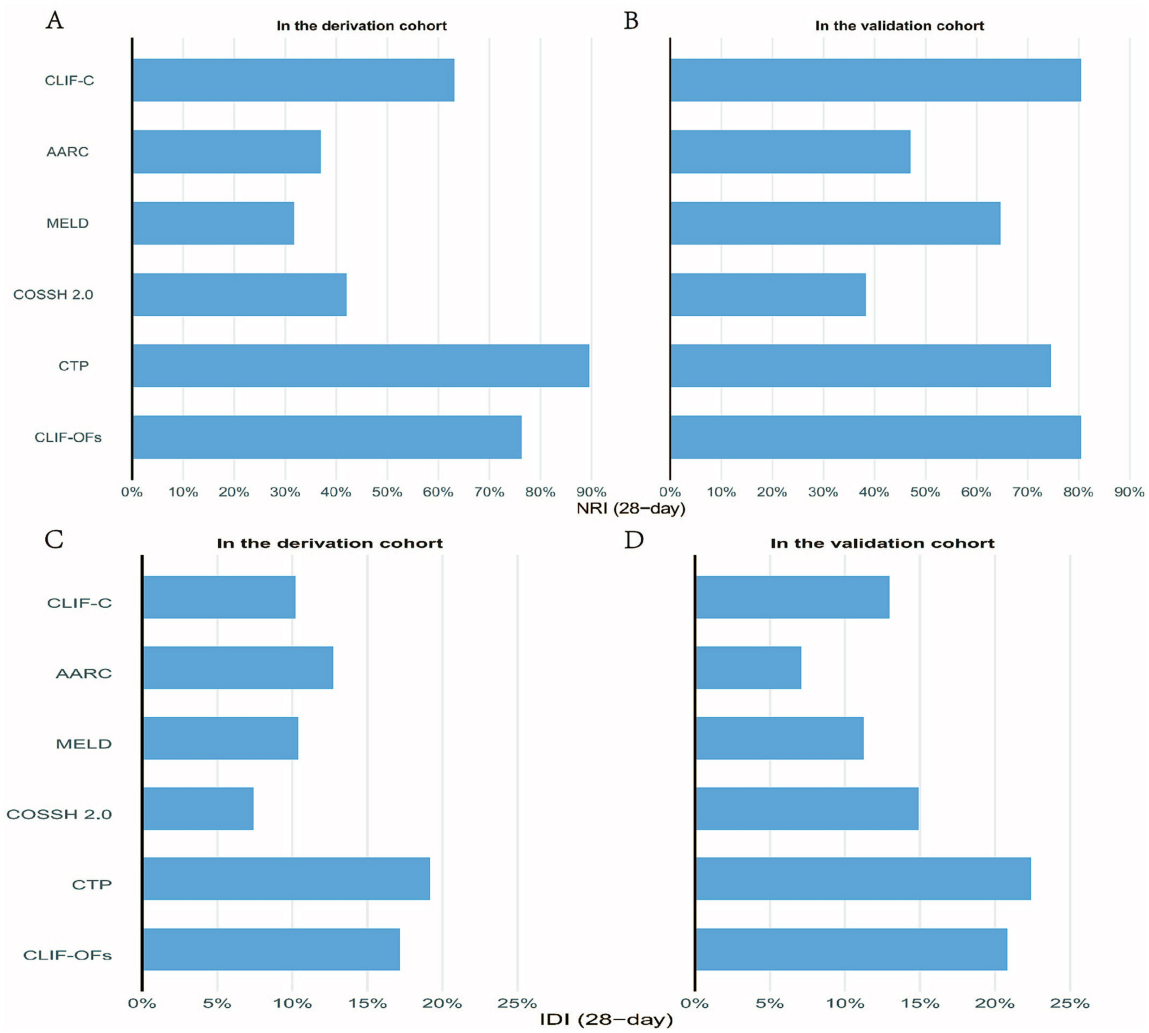
NRI and IDI for model comparison. Panels **(A,B)** show the net reclassification improvement (NRI) of the ICI and traditional models in the training and validation sets, respectively. Panels **(C,D)** display the integrated discrimination improvement (IDI) for the same models in the training and validation sets.

**Figure 7:**
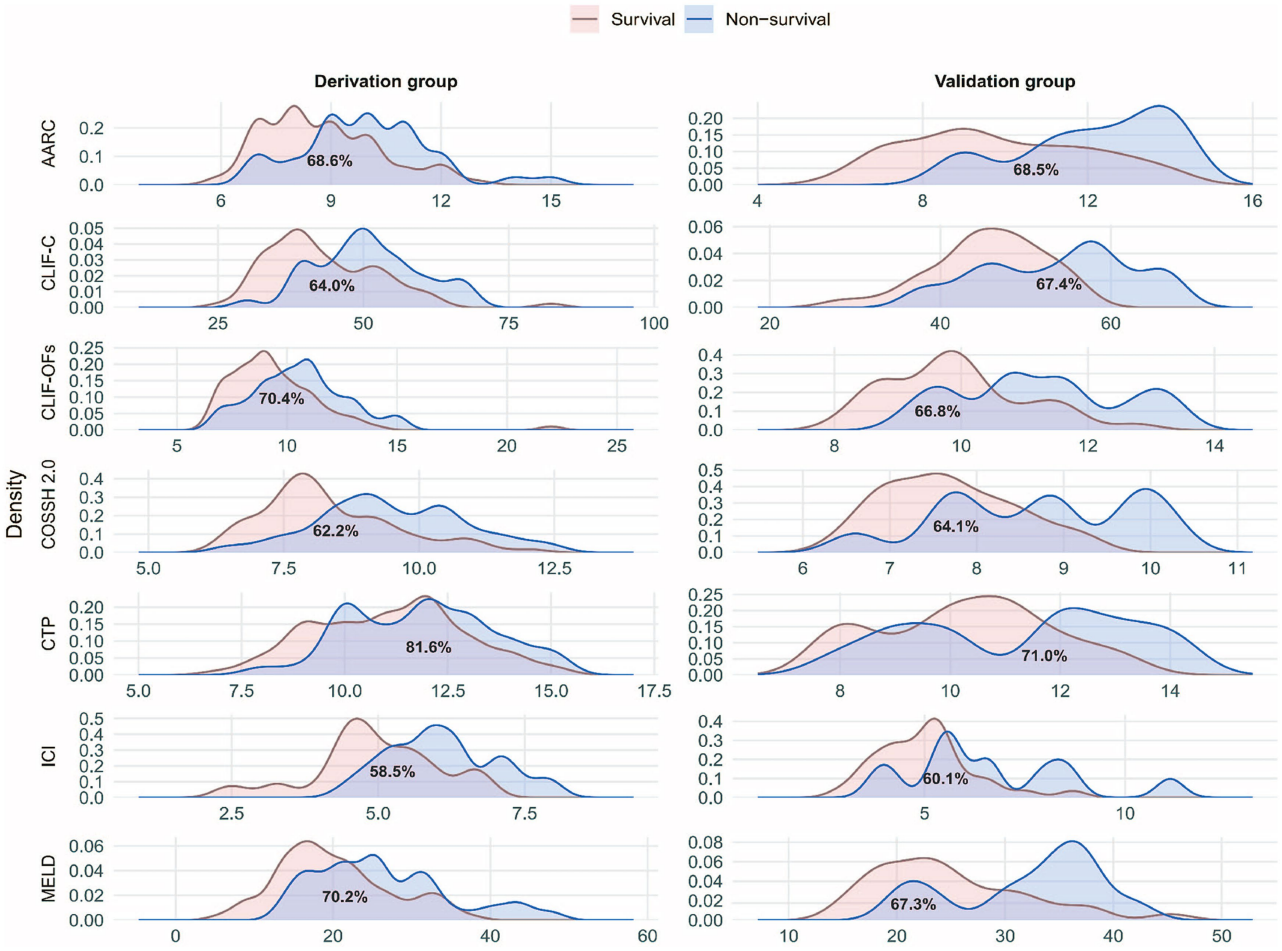
Probability density and overlap of prognostic scores. Panels show the probability density of raw prognostic scores for surviving and non-surviving patients in the derivation and validation cohorts. The grey ribbon indicates the overlap between the two groups, with the overlap percentage (OVL%) displayed at the centroid.

By comparing the ICI model with six commonly used ACLF scoring systems, we further validated the superiority of the new model. ROC curve analysis (Figures 5A,B) showed that the ICI model demonstrated significantly higher AUCs than traditional models such as COSSH-ACLF II (0.744/0.712), CLIF-C (0.734/0.707), and MELD (0.706/0.682) in both the training and validation cohorts. The DeLong test results (Table 3) indicated that the differences in AUC between the ICI model and all traditional models were statistically significant (*p* < 0.05) in both cohorts, suggesting that the ICI model has strong discriminatory ability and stable predictive performance. Moreover, Net Reclassification Improvement (NRI) and Integrated Discrimination Improvement (IDI) (Figure 6) showed that the ICI model outperformed traditional models in terms of reclassification ability and predictive accuracy. According to PDF analysis (Figure 7), the overlap coefficient of the ICI model in the training and validation cohorts was 58.5 and 60.1%, respectively, significantly higher than that of other traditional models, such as AARC (68.6%/68.5%), CLIF-C (64.0%/67.4%), COSSH-ACLF II (70.4%/66.8%), CTP (81.6%/71.0%), and MELD (70.2%/67.3%). This indicates that the ICI model has superior discriminatory power in predicting 28-day survival. Finally, the clinical decision curve results demonstrated that the ICI model provides better clinical benefit compared to traditional models (Figures 5C,D).

**Table 3:** DeLong test results for comparing ROC curves.

Comparison	*P*-value (Training set)	*P*-value (Test set)
ICI vs. CLIF-C	0.0406	0.0286
ICI vs. AARC	0.0132	0.0406
ICI vs. MELD	0.0059	0.0069
ICI vs. COSSH 2.0	0.0438	0.0358
ICI vs. CTP	0.0001	0.0025
ICI vs. CLIF-OFs	0.0054	0.0473

The table displays the results of the DeLong test, which compares the AUC values between different models.

## Discussion

Early identification of high-risk patients with HBV-ACLF is essential for improving clinical outcomes, as it not only supports timely treatment decisions but may also reduce the substantial short-term mortality associated with the disease (19, 21). In this study, we developed and validated a concise and practical nomogram—the ICI model—based on three routinely available indicators: INR, Cr, and IL-6. This model was designed to predict 28-day mortality in patients with HBV-related ACLF. The ICI model demonstrated good discriminative ability and calibration consistency in both the training and external validation cohorts. Decision curve analysis further confirmed its superior net clinical benefit across a wide range of clinically relevant threshold probabilities. Compared with conventional scoring systems, the ICI model exhibited advantages in both predictive performance and accessibility of variables.

Currently, prognostic scoring systems such as MELD, CLIF-C, and COSSH-ACLF II are widely used for risk stratification in patients with ACLF. However, due to the lack of a unified definition of ACLF, these models differ significantly in terms of development background, variable composition, and target populations, and a universally applicable standardized model remains unavailable (22, 23). For example, the MELD score is structurally simple and based on total bilirubin, INR, and creatinine (14), and was originally developed for organ allocation in end-stage liver disease (24). However, studies have indicated that MELD has limited accuracy in predicting short-term mortality in ACLF patients (17, 25). The CLIF-C series, derived from the CANONIC study, focuses on multi-organ failure scoring and is primarily suited for alcohol-related ACLF in European populations (16). Nonetheless, its complexity and reduced performance in HBV-ACLF limit its applicability in HBV-endemic regions (16, 17, 26). The COSSH-ACLF scoring systems were developed from a large Chinese HBV cohort and demonstrate good predictive performance within this population. However, because their diagnostic criteria emphasize severe hepatic injury and organ failure, their ability to detect early, subclinical deterioration is limited, which affects generalizability (16, 18). The AARC score, derived from an etiologically mixed cohort, may not adequately capture the distinct progression patterns of HBV-ACLF, thereby restricting its utility in this specific subgroup (2).

An increasing body of evidence recognizes ACLF as an inflammation-driven syndrome, characterized not only by hepatic parenchymal injury but more importantly by systemic immune activation that leads to a cascade of multi-organ failure (27–31). Acute triggers such as infections or drug-induced liver injury can provoke an overwhelming immune response, marked by a rapid surge in pro-inflammatory cytokines like IL-6 and TNF-*α*. This “cytokine storm” contributes to microcirculatory dysfunction and extrahepatic organ impairment (12, 32). Among these cytokines, IL-6 has shown strong correlations with short-term mortality in ACLF patients and is emerging as a valuable prognostic biomarker (33–35). Beyond its role as a classical inflammatory mediator, IL-6 exerts pathogenic effects through the JAK/STAT3 signaling pathway, promoting monocyte–macrophage chemotaxis, upregulation of adhesion molecules, and secondary cytokine cascades that amplify tissue injury (36, 37). Moreover, IL-6 has been shown to impair mitochondrial function and disrupt cellular bioenergetics, contributing to immune cell exhaustion during ACLF progression (38–40). Therapeutic antibodies targeting the IL-6/IL-6R, such as tocilizumab, have demonstrated long-term efficacy and safety in diseases like rheumatoid arthritis and COVID-19 (41–43). These findings suggest that targeting IL-6-mediated pathways could offer a promising strategy for precision treatment in ACLF. In our study, IL-6 was incorporated into the prognostic model not only to reflect its central role in ACLF pathogenesis but also to improve the identification of “subclinical inflammatory risk phenotypes” that are often overlooked by traditional scoring systems.

The ICI model integrates a mechanism-oriented and data-driven approach by incorporating three key variables — INR, Cr, and IL-6 — to construct a predictive framework that reflects hepatic synthetic capacity, renal compensatory function, and systemic inflammatory status. This design improves the identification of patients at risk for early progression. INR and Cr are already core components of several established prognostic scores, highlighting their clinical significance in ACLF. Specifically, INR quantifies coagulation function and serves as a surrogate marker for hepatic synthetic function in patients with liver disease. Cr is a key indicator for assessing renal function. Its elevation is often associated with decreased renal perfusion caused by systemic inflammation and haemodynamic disturbances, acute kidney injury (AKI) or even haemorrhagic renal syndrome (HRS), even mild elevations are significantly associated with increased mortality (44, 45). IL-6, as a representative marker of systemic inflammation, further enhances the mechanistic interpretability of the model. Its pivotal role in the pathogenesis of ACLF has been discussed previously. In comparison with complex models that rely on multi-organ failure scoring systems, the ICI model features a simplified structure and strong practicality, demonstrating promising potential for clinical translation.

Because inflammatory biomarker selection directly affects a model’s interpretability and reproducibility, we performed a systematic comparison of candidate inflammatory biomarkers and conducted sensitivity analyses to evaluate how key variable choices influenced overall model performance, using data from the training cohort (Supplementary Tables S1,S2). In univariate analyses, ln(IL-6) showed a stable positive association with 28-day mortality (O*R* = 2.113, 95% CI 1.398–3.194; *p* < 0.001) and demonstrated the strongest overall discrimination among candidate biomarkers (AUC = 0.716, 95% CI 0.615–0.817; DeLong comparisons in Supplementary Table S1). Notably, ln(WBC) was also associated with mortality (O*R* = 3.637, 95% CI 1.674–7.903; *p* = 0.001), with an AUC of 0.696 (95% CI 0.589–0.803), which did not differ significantly from that of ln(IL-6) (DeLong *p* = 0.771). ln(WBC) was also retained as a candidate predictor during initial LASSO screening. To quantify the incremental predictive value of ln(WBC), we further developed and compared candidate models with and without ln(WBC) (Supplementary Table S2). The model including ln(WBC) showed discrimination comparable to the final ICI model (AUC: 0.833 vs. 0.826; DeLong *p* = 0.421) with nearly identical prediction error (Brier score: 0.160 vs. 0.162). Adding ln(WBC) to the ICI model did not significantly improve model fit (LRT *p* = 0.335), and information criteria (AIC/BIC) favored the more parsimonious model. Moreover, the model substituting ln(WBC) for ln(IL-6) showed overall inferior performance (AUC: 0.787; Brier score: 0.174; higher AIC/BIC than the ICI model), although the AUC difference versus the ICI model was not statistically significant (DeLong *p* = 0.237). Importantly, a non-significant DeLong test (*p* > 0.05) does not imply equivalence or interchangeability. Considering discrimination, prediction error, and model parsimony together, ln(IL-6) was prioritized as the inflammation-related predictor in the final model.

While these analyses serve to strengthen the evidence base for variable selection and model simplification, it is important to note that several limitations of the study remain. Firstly, the sample size and the source of the validation cohort are relatively limited, and further validation of the model’s stability is required using multicentre data from heterogeneous populations. Secondly, the model was constructed based on a single time point at admission and does not account for the trend of changes in the indicators. It is recommended that subsequent studies investigate the potential for the model framework to be updated in a dynamic manner. Thirdly, the impact of the model on actual clinical decision-making (e.g., ICU admission, artificial liver support, liver transplantation prioritization) has not yet been evaluated, and prospective studies are needed to validate its clinical utility.

## Conclusion

In summary, the ICI model demonstrates excellent performance in terms of mechanistic rationality, predictive performance and clinical feasibility. It is anticipated that the ICI model will serve as a valuable instrument in complementing the conventional scoring system for the HBV-ACLF population in the future.

## Data Availability

The raw data supporting the conclusions of this article will be made available by the authors, without undue reservation.
